# Molecular surveillance and predictive risk modelling of avian influenza virus in wild birds in Egypt

**DOI:** 10.1099/jgv.0.002278

**Published:** 2026-06-16

**Authors:** Nehal M. Nabil, Maram M. Tawakol, Naglaa Hagag, Samah Eid, Liam Brierley, Mahmoud M. Naguib

**Affiliations:** 1 Reference Laboratory for Veterinary Quality Control on Poultry Production, Animal Health Research Institute, Agriculture Research Centre, Giza, Egypt; 2 MRC-University of Glasgow Centre for Virus Research, University of Glasgow, Glasgow, UK; 3 Department of Infection Biology and Microbiomes, Institute of Infection, Veterinary and Ecological Sciences, University of Liverpool, Liverpool, UK; 4 Zoonosis Science Centre, Department of Medical Biochemistry and Microbiology, Uppsala University, 751 21 Uppsala, Sweden

**Keywords:** Avian influenza virus, Egypt, machine learning, migratory birds, spatial risk modelling

## Abstract

Avian influenza viruses (AIVs) continue to circulate and pose a persistent threat to animal and public health in Egypt, a country located along major migratory birds’ flyways that enable repeated viral incursions. In this study, a total of 1,087 wild birds representing 19 species were sampled to assess the role of migratory wild birds in virus introduction and spread. Six species were found positive for AIV, with Eurasian teal showing the highest prevalence (12.3%). The H5 subtype was found predominant, representing 70.3% of positive cases, whereas H9 was detected in 9.9% of positive cases. Moreover, coinfections with H5/H9 were observed in Eurasian teal, Common moorhen and Northern pintail, suggesting opportunities for cocirculation among migratory species. To predict areas at high risk of AIV detection, these findings were integrated with additional publicly available geolocated AIV sampling records and a spatial machine learning (ML) model was trained. The model demonstrated moderate predictive performance (Area Under Curve =0.608; F1=0.596) and highlighted relative humidity, temperature and proximity to wetlands as primary predictors of AIV detection. In addition, risk projections for early winter underscore several areas including the Nile Delta, Nile River corridor, southern wetlands near Lake Nasser, the south-eastern Red Sea coast and the north-western Siwa region as areas of high risk of AIV detection. The findings of this study demonstrate the role of migratory wild birds in the spread and introduction of AIV into Egypt, particularly H5, and provide an ML-based model to predict areas of high risk of virus detection in Egypt, which could in turn strengthen early detection and guide mitigation strategies.

## Introduction

Avian influenza viruses (AIVs) continue to circulate globally in wild avian species and spread to infect poultry and a range of mammalian species including humans [1,2]. Based on the antigenic and genetic properties of the haemagglutinin (HA) and neuraminidase surface proteins, AIVs are classified into H1-16, H19 and N1-9 subtypes [3]. Furthermore, according to their pathogenicity and disease severity in chickens, AIVs can be classified into high pathogenicity avian influenza (HPAI) virus and low pathogenicity avian influenza (LPAI) virus [4].

The ability of wild birds, in particular orders Anseriformes (mainly ducks, geese and swans) and Charadriiformes (mainly gulls, terns and waders), to maintain a high diversity of AIV and to be highly mobile facilitates the emergence and spread of new strains of AIV. Migratory bird movements have been associated with the long-distance spread of HPAI H5Nx viruses [5], including the global spread of clade 2.2.1 HPAI H5N1 virus between 2005 and 2007 [6], the HPAI H5N8 virus outbreaks by clade 2.3.4.4b in 2016–2017 [7] and the recent wave of HPAI H5N1 virus panzootic of clade 2.3.4.4b in 2023–2025 [8]. Recent reports indicate the wide spread of this clade in Asian countries, including China, Cambodia and Bangladesh [9,11] where multiple strains have been isolated from domestic and wild birds, highlighting its continued circulation and diversification in the region. Wild birds can affect AIV epidemiology and evolution in poultry locally through the emergence of new AIV subtypes/genotypes via reassortment and incursions into new areas. Once the virus is introduced and adapted to poultry, viruses are able to keep circulating among poultry populations.

Egypt acts as a bridging location between Africa and Eurasia [12], evident in the migratory birds flyways, where Egypt serves as a link between the Palearctic with the Afro-Tropical regions. In Egypt, the Mediterranean–Black Sea and East Africa–West Asia flyways intersect with the more regional Rift Valley–Red Sea flyway [13]. Along the Rift Valley–Red Sea flyway, migratory birds, particularly bird species from order Anseriformes and Charadriiformes, from Europe and Asia fly down the eastern Mediterranean coast or the Jordan Valley to northern Egypt before wintering along the Mediterranean coast, Nile Valley or Red Sea coast, or continue southwards towards the East African Rift Valley and Sub-Saharan Africa.

In Egypt, the first reported HPAI H5N1 virus of clade 2.2.1 was originally documented from an Eurasian green-winged teal trapped in a cage by a fisherman in the Damietta governorate of northern Egypt in December 2005 [14]. Later in 2016 and in the same governorate, HPAI H5N8 virus was detected in wild bird [common coots (*Fulica atra*)] in 2016 [15], followed by HPAI H5N8 virus detection in two apparently healthy Eurasian green-winged teals sampled in a live bird market (LBM) in Port Said in northern Egypt early December 2016 [16]. Here, we investigate the prevalence of AIV in a variety of wild bird species in Egypt and provide a machine-learning risk model to predict areas of high risk of AIV detection in Egypt.

## Methods

### Sample collection

**Fig. 1.**
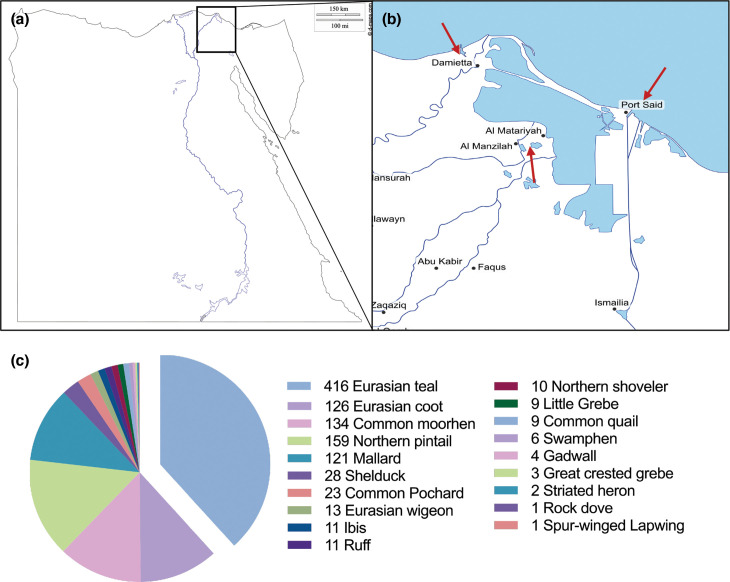
Sites of samples collection and total number of samples per bird species.

Oropharyngeal and cloacal swab samples from 1,087 migratory birds were obtained during 2019–2023. Samples were collected from 19 different bird species as shown in Fig. 1 and Table S1, available in the online Supplementary Material. Sampling was conducted during the autumn–winter period (September to January), corresponding to peak migration and overwintering seasons in Egypt. Birds were sampled in four different geographical locations near water sources at the north part of Egypt including Damietta, Port Said and Dakahlia (Al Manzilah and Al Matariyah) governorates (Fig. 1). Samples were pooled by species, date of collection and sampling location; each pool consisted of five to ten individual swabs collected from the same bird species at the same site on the same day.

### Molecular detection and virus isolation

Briefly, viral RNA from each pooled sample was extracted using the QIAamp Viral RNA Mini Kit (Qiagen, Hilden, Germany) according to the manufacturer’s instructions. All extracted RNAs were initially examined for the matrix (M) gene of influenza A viruses using standard quantitative reverse transcription polymerase chain reaction (RT-qPCR) specific [17]. Positive influenza M RNAs were then screened using gene-specific RT-qPCR assays for the HA gene segment of the AIV H5 and H9 [18]. The RT-qPCR reactions were conducted using Stratagene MX3005P real-time PCR machine (Agilent, Santa Clara, CA, USA). A sample was considered positive for AIV by RT-qPCR when the cycle threshold (Ct) value was <38. Sequence of the primers used in these assays are provided in Table S2

### External AIV detection data and predictor variables

To model risk of AIV detection, we sourced additional geolocated and dated sampling data for wild birds reported within country boundaries of Egypt from the Bacterial and Viral Bioinformatics Resource Center (BV-BRC) [19], Emergency Prevention System Global Animal Disease Information System (EMPRES-i) [20] and World Animal Health Information System (WAHIS) [21]. Both positive and negative sampling records are available from BV-BRC, although EMPRES-i and WAHIS provide only positives. To maximize data availability for model training, all LPAI and HPAI records were included, regardless of subtype. This gave an additional 7,710 raw records spanning 2006–2019.

**Fig. 2.**
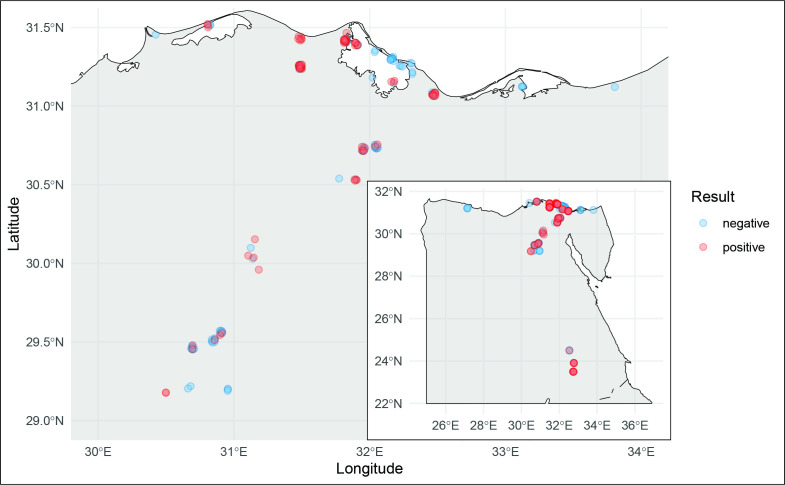
A total of 219 unique instances of geolocation–calendar date combinations for avian influenza sampling in wild birds in Egypt. Main figure depicts the north-east region, and inset depicts several additional instances across wider Egypt. Colour denotes sampling result. Points have been geographically jittered to prevent overlap and show density of instances.

Instances of unique calendar date–geolocations were considered as the base unit for models, taking positives as calendar date–geolocation combinations with at least one positive sample (regardless of specific species or year), and negatives as those sampled combinations only ever reporting negative results. This resulted in 94 positive instances and 125 negative instances across 46 geolocations total (Fig. 2).

Following Hayes *et al*. [22], a suite of environmental and landscape-level variables was extracted for each sampling instance to train a predictive model for the presence of avian influenza: distance to the nearest coast, distance to the nearest inland water (permanent water bodies only) [23], elevation [24], land cover type (17 possible categories) [25], poultry population (both chicken and duck counts) [26], Normalized Difference Vegetation Index (NDVI) for the calendar date (within a 16-day period) [27], and several bioclimatic variables each calculated over the 30-day period prior to the calendar date of the sample instance: mean relative humidity, mean temperature, mean diurnal temperature range, total precipitation and minimum elevation of zero-degree isotherm [28]. Nearest-neighbour values were assigned in cases where geolocations had missing variables. Detailed spatial model predictors are provided in the supplementary method file.

### Machine learning prediction of risk of AIV detection in Egypt

We followed a species distribution modelling approach, an ecological model type for predicting habitat suitability for terrestrial species, although equivalent methods can be applied to map distributions of diseases [29]. We trained Bayesian additive regression tree (BART) to predict AIV presence using R package ‘embarcadero’ v1.2.0.1003 [30]. As a tree-based method, BART can capture non-linear interactions among predictors and allows for intuitive calculation of uncertainty around predictions within its Bayesian framework.

Data instances were divided into training and test sets at a 3 : 1 ratio. As geolocations in close proximity are likely to have similar predictor values and AIV status, those within a Euclidean distance of 0.15° were grouped together for training/test set assignment. Training data were then augmented using random over-sampling examples (ROSE) methods [31] via package ‘ROSE’, v0.0–4 [32]. BART models were trained prioritizing classification of positive instances at a 2 : 1 ratio. Model parameters were optimized via fivefold cross-validation and stepwise variable selection was conducted, before validating predictive power of models on the held-out test set.

The final best-performing model was further explored by comparing importance of each predictor variable by calculating standardized frequency of use in model decision points. To infer AIV risk across wider areas of Egypt, we generated and visualized model predictions over the full country area at a 0.1-degree grid resolution. Predictions were made for two seasonal calendar points to represent winter and summer conditions (January 1 and July 1, respectively) in bioclimatic predictors. All analyses were conducted using R version 4.5.1.

## Results

### High prevalence of H5 in anatidae and rallidae

**Fig. 3.**
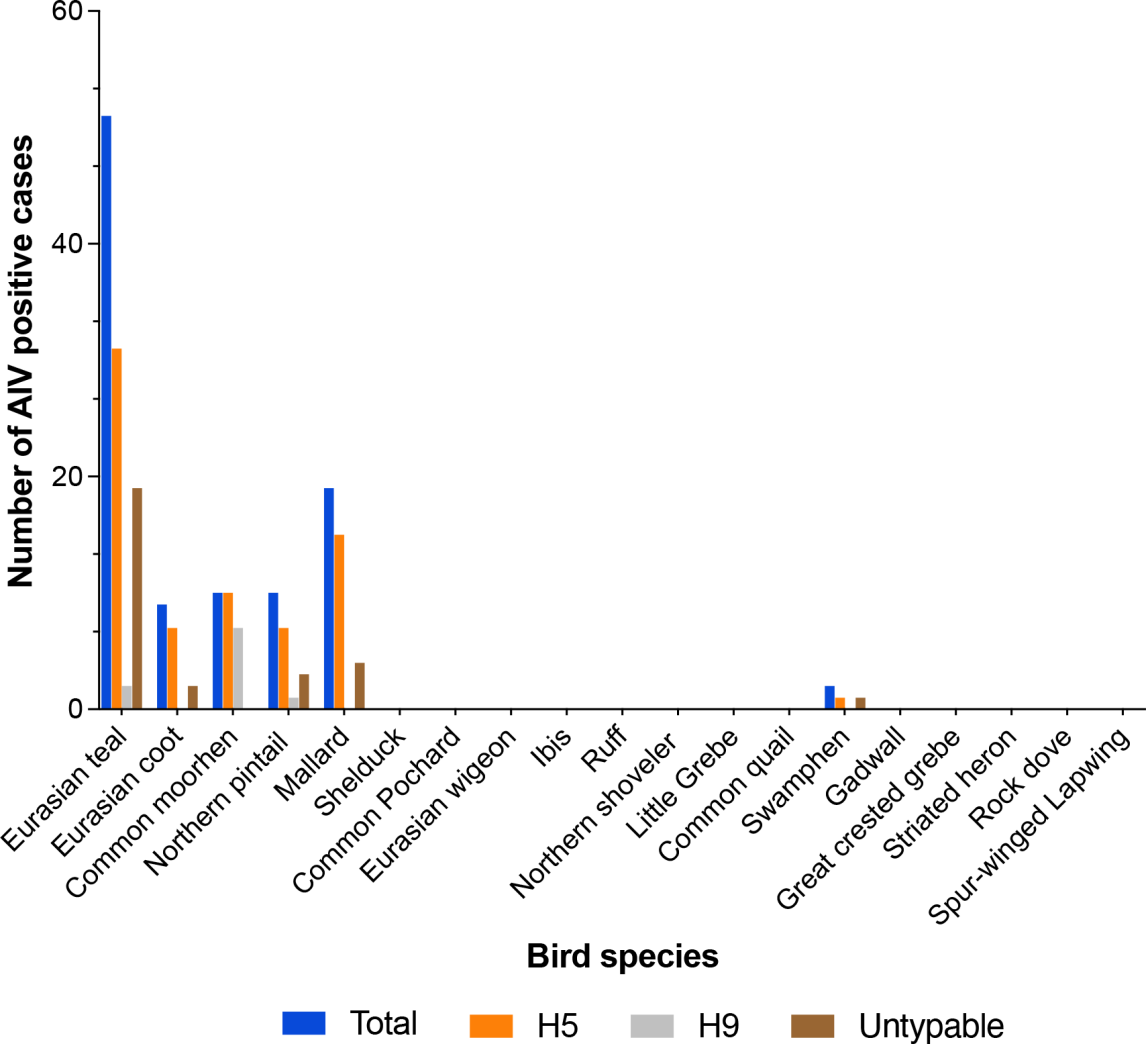
Number of influenza A/H5- and A/H9-positive cases in different samples collected in this study.

A total of 1,087 wild birds, representing 19 species, were sampled for the detection of AIV virus and further subtyping for subtypes H5 and H9. Six species were examined positive for AIV, while the remaining 12 species were negative for both H5 and H9 subtypes (Fig. 3).

The Eurasian teal (*Anas crecca*) was the most commonly sampled species (*n*=416), with 51 (12.3%) positive cases for AIV. Among these positives, 31 (60.8%) were tested positive for H5 subtype, only 2 (3.9%) for the H9 subtype and 19 (37.3%) were negative for both H5 and H9 subtypes (Fig. 3 and Table S1). The Eurasian coot (*F. atra*) showed 9 positives (7.1%) out of 126 collected samples, 7 positives for H5 and none for H9 (Fig. 3).

Overall, H5 subtype was the predominant subtype and detected in 71 sampled birds (70.3%), while H9 subtype was detected in 10 birds (9.9%). Mixed infections with both H5 and H9 subtypes were observed primarily in Common moorhen (*n*=7), Eurasian teal (*n*=2) and Northern pintail (*n*=1).

### Predictive model capacity for AIV detection

We combined our sampling results with additional geolocated AIV data drawn from open animal disease data platforms, giving 219 unique geolocation–calendar date combinations of sampling instances. These were largely concentrated towards north-eastern Egypt, with the greatest density of instances around Damietta and Port Said (Fig. 2).

When a predictive BART machine learning (ML) algorithm was trained on these data, final model performance on held-out test data was adequate (Area Under Curve (AUC)=0.608, F1 score=0.596) with slightly stronger ability to predict negative samples (sensitivity=0.567, specificity=0.706).

**Fig. 4.**
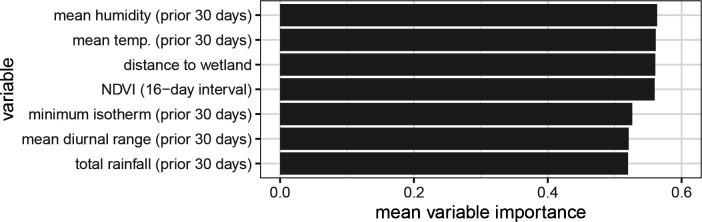
Importance of each variable used in final predictive ML model. X axis depicts mean frequency of variable appearance over all individual decision tree models sampled from the BART ensemble.

Following stepwise variable selection, this model discriminated between positive and negative instances of AIV using only seven predictors, most of which were bioclimatic (Fig. 4). Mean relative humidity, mean temperature, vegetation cover and proximity to wetland appeared slightly more influential in model decisions than other bioclimatic variables (Fig. 4). Neither land geography (e.g. elevation, land cover type) nor poultry population were used in the final model.

### Model projections of AIV risk across Egypt

**Fig. 5.**
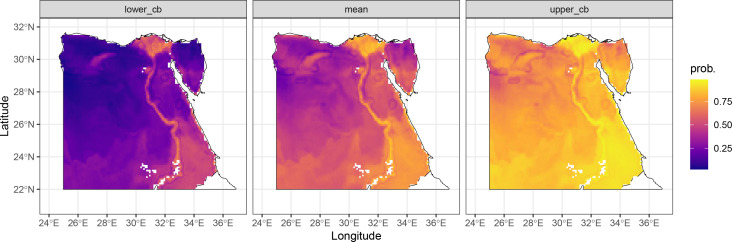
Projected probability of avian influenza presence over Egypt from final BART ML model for calendar date of January 1. Mean probability is shown, as well as upper and lower credible bounds representing 95% interval of posterior probabilities*.*

This model was used to project predicted AIV risk across Egypt for a sampling calendar date of January 1 within the winter bird migratory season (Fig. 5). Areas with higher risk included the Nile River and delta in the north, the south-eastern region, and areas of the north-west from the Qattara Depression to Siwa lakes. Model uncertainty suggested waterways of the Nile to be predicted most confidently as having AIV positives, and areas towards the western border to be predicted most confidently as having negatives. Projected risk was much more uniform for summer predictions, likely reflecting the more homogenous climate throughout Egypt and lack of bird migration (Fig. S1).

## Discussion

AVI has remained endemic in Egypt for 20 years, since the first detection of AIV H5 in December 2005 [33]. Multiple introductions of HPAI H5 viruses into Egypt, representing multiple subtypes and genotypes, have been linked to migratory wild birds. For example, HPAI H5N1 and H5N8 viruses were first identified in migratory waterfowl at northern stopover sites such as Damietta [34,35] and subsequently spilled over into domestic poultry resulting in widespread across over Egyptian poultry holdings [34].

The present study reports the prevalence patterns of AIV in wild migratory birds sampled from key wetlands in Egypt. Among the 1,087 samples representing 19 species of wild birds, a high number of AIV-positive cases were observed in waterbird species, particularly the Eurasian teal (*A. crecca*). The high prevalence of the H5 subtype aligns with previous reports [13] and emphasizes the role of wild migratory birds in virus spread and incursion of new viral genotypes or lineages into Egypt. Additionally, few cases of H5/H9 coinfections were detected in Common moorhen, Eurasian teal and Northern pintail, indicating the possibility for viral reassortment and the emergence of new strain(s), although such events were not directly assessed in this study.

ML modelling has become an important tool for predicting areas at high risk of AIV detection [22,36, 37]. The models can integrate large and heterogeneous datasets such as wild bird migration patterns, poultry density, environmental variables and climate data to map spatial AIV risk and enable earlier detection and targeted surveillance. To better understand climatic and environmental drivers associated with AIV detection and predict areas of high risk of virus detection in Egypt, this study integrated the results of collected samples with publicly available records and trained a BART predictive model. Sparse data and the diffuse nature of AIV spread mean that likely detection locations are a challenge to predict with high precision. However, some generalities are evident from our trained ML model. AIV was more likely to be present in humid and highly vegetated conditions closer to wetlands. These sites have been recognized by BirdLife International as stopover and wintering locations for wild migratory birds, which supports the model’s biological credibility [38]. This includes the Nile delta which has received moderate surveillance efforts (especially the low wetlands around Port Said), but the presented model also highlighted additional high risk with minimal historical surveillance areas including the southern region around Lake Nasser and the south-east coast as potentially important sites for AIV in wild birds. In addition, the north-west around Siwa lakes which was described previously as one of the main stop sites of wild migratory birds in Egypt [39]. Geographically, the model’s highest confidence occurred along the Nile River, where the probability of detecting positives was consistently high, while negative predictions with high certainty were found in the western deserts due to the absence of suitable wetland habitat (Fig. 5).

Our spatial ML model predicted AIV risk from environmental variables. While physical conditions can determine virus stability and persistence [40], we expect most of the model’s predictive power approximates changing density of wild migratory birds. Other spatial model studies have successfully applied predictors to directly measure wild bird density and/or diversity [22,37]. Unlike these European studies, however, we were not able to compare environment against avian host predictors due to a lack of coverage and quality of spatial wild bird data for Egypt. Further in-depth surveys of Mediterranean species and how their density changes around Egyptian waterways over the calendar year are likely necessary to improve the moderate predictive performance of the models presented here, particularly considering the homogeneous risk patterns for warmer seasons (Fig. S1).

Taken together, our findings emphasize the central role played by wild migratory birds in the introduction and dissemination of AIV viruses. The environmental variables highlighted by our model including humidity, temperature and wetland provide a foundation for anticipating areas of high risk of virus detection and understanding how changing climatic conditions may reshape AIV ecology. In addition, the ML-based models provided in this study can be implemented to guide surveillance strategies by identifying overlooked high-risk areas. In conclusion, this study combines field surveillance and ML approaches to advance our understanding of AIV risk across Egypt and calls for continuing surveillance across multiple migratory species, particularly those closely associated with wetlands.

## Supplementary material

10.1099/jgv.0.002278 Supplementary Material 1.

## Abbreviations

AIV: avian influenza virus
AUC: Area Under Curve
BART: Bayesian additive regression tree
BV-BRC: Bacterial and Viral Bioinformatics Resource Center
EMPRES-I: Emergency Prevention System Global Animal Disease Information System
HA: haemagglutinin
HPAI: high pathogenicity avian influenza
LBM: live bird market
LPAI: low pathogenic avian influenza
NDVI: Normalized Difference Vegetation Index
ROSE: random over-sampling example
RT-qPCR: reverse transcription polymerase chain reaction
