# Supplementary material for: Molecular surveillance and predictive risk modelling of avian influenza virus in wild birds in Egypt
Source: J Gen Virol. 2026 Jun 16;107(6):002278. doi: 10.1099/jgv.0.002278 (PMC13271260; doi:10.1099/jgv.0.002278)
Supplement: Supplementary Material 1. [file jgv-107-02278-s001.pdf]

Supplementary Materials:

Supplemental Figure 1. Projected probability of avian influenza presence over Egypt from final BART machine learning model for calendar date of July 1.

Mean probability is shown, as well as upper and lower credible bounds representing 95% interval of posterior probabilities.

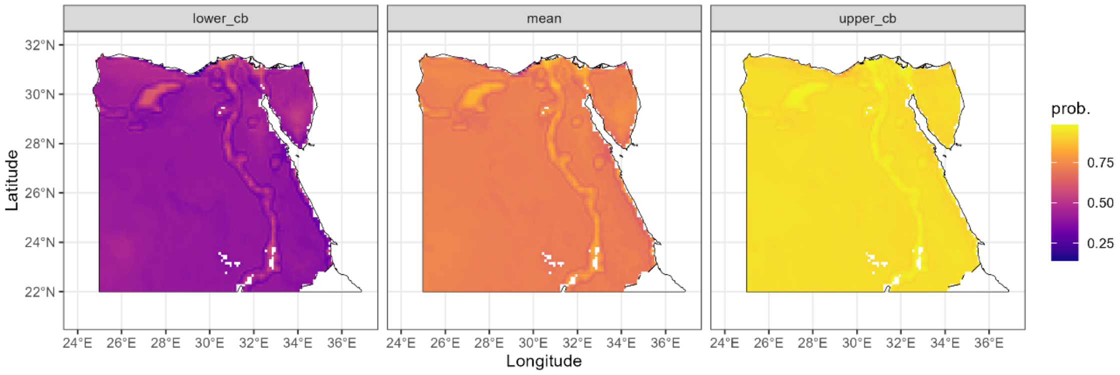

Supplementary Table 1. Bird species and influenza A virus (IAV) positive cases

Name	Scientific name	Number of samples	IAV positive	H5	H9	not H5 not H9
Eurasian teal	*Anas crecca*	416	51	31	2	*19*
Eurasian coot	*Fulica atra*	126	9	7	0	*2*
Common moorhen	*Gallinula chloropus*	134	10	10	7	0
Northern pintail	*Anas acuta*	159	10	7	1	3
Mallard	*Anas platyrhynchos*	121	19	15	0	*4*
Shelduck	*Tadorna*	28	0	0	0	*0*
Common Pochard	*Aythya ferina*	23	0	0	0	*0*
Eurasian wigeon	*Mareca penelope*	13	0	0	0	*0*
Ibis	*Threskiornithinae*	11	0	0	0	*0*
Ruff	*Calidris pugnax*	11	0	0	0	*0*
Northern shoveler	*Spatula clypeata*	10	0	0	0	*0*
Common quail	*Coturnix coturnix*	9	0	0	0	*0*
Swamphen	Porphyrio	6	2	1	0	*1*
Gadwall	*Anas strepera*	4	0	0	0	*0*
Great crested grebe	*Podiceps cristatus*	3	0	0	0	*0*
Striated heron	*Butorides striata*	2	0	0	0	*0*
Rock dove	*Columba livia*	1	0	0	0	*0*
Spur-winged Lapwing	*Vanellus spinosus*	1	0	0	0	*0*
Little Grebe	*Tachybaptus ruficollis*	9	0	0	0	*0*
		**1087**	**101**	71	10	29

Supplementary Table 2. Primers used for RT-PCR

Label	Sequence	Reference
Avian Influenza Virus M RT-qPCR		(Spackman, 2020)
AIV forward	5’ - AGATGAGTCTTCTAACCGAGGTCG - 3’	
AIV reverse	5’ - TGCAAAAACATCTTCAAGTCTCTG - 3’	
AIV probe	5’ - [FAM]TCAGGCCCCCTCAAAGCCGA[BHQ-1] - 3’	
H5 specific RT-qPCR		(Hassan et al., 2022)
H5-HA1-F	5’ - GATTYTAAARGATTGTAGYGTAGC - 3’	
H5-F2	5’ - GTTCCCTAGYAYTGGCAATCAT - 3’	
H5-HA-R1	5’ - CTCTCYACCATGTARGACCA - 3’	
H5-HA-R2	5’ - CTCTCYACTATGTARGACCA - 3’	
H5-R3	5’ - AATTCTARATGCAAATTCTGCAYTG - 3’	
H5-FAM3-RC	5' - [6-FAM]CGCACATTGGRTTYCCRAGGAGCC[BHQ-1] - 3’	
H5-FAM2	5' - [6-FAM]CTGGTCTATYYTTRTGGATGTGCTCC[BHQ-1] - 3’	
H9 specific RT-qPCR		(Hassan et al., 2022)
H9 forward 1	5’ - CAATGGGGTTYGCTGCCT - 3’	
H9 forward 2	5’ - CAATGGGRKTTGCTGCCT - 3’	
H9 reverse	5’ – TTATATACARATGTTGCAYCTG - 3’	
H9 probe	5’ - [FAM]TTYTGGGCCATGTCIAATGGRTC[BHQ-1] - 3’	

Supplementary Methods
Spatial model predictors

Following a previous spatial machine learning approach for avian influenza in wild birds (Hayes et al., 2025), we derived several environmental predictor variables for each avian influenza sampling geolocation (or geolocation-calendar date instance, for time-variant, seasonal predictors). Distance to nearest coast was calculated using spatial vector maps from the Natural Earth dataset obtained via R package `rnaturalearth`, v1.1.0 (Massicotte and South, 2025). Distance to nearest inland water was calculated with reference to permanent inland water bodies as defined by Level 3 of the Global Lakes and Wetlands Database from the World Wildlife Fund (Lehner and Döll, 2004). Elevation was sourced from the CGIAR-Shuttle Radar Topography Mission (SRTM) dataset (Jarvis et al., 2008), obtained using the `elevation_global` function in R package `geodata`, v0.6-2 (Hijmans et al., 2024).

Land cover was sourced from Moderate Resolution Imaging Spectroradiometer (MODIS) dataset MCD12Q1 v6.1 taking the most recent year of data availability (2022) (Friedl and Sulla-Menashe, 2022). This data classifies land type into 17 categories, although only the following 6 were represented within avian influenza sampling geolocations in Egypt: savanna, grassland, permanent wetland, cropland, non-vegetated barren land, and water body. Normalised Difference Vegetation Index (NDVI) was sourced from MODIS dataset MOD13Q1 v6.1 taking the most recent year of data availability (2022) (Didan, 2022). Geolocated sampling instances were assigned values based on matching their calendar date to data periods of NDVI data (available at 16-day intervals). Both land cover and NDVI data were obtained using the `getNASA` function in R package `luna`, v0.3-7 (Hijmans and Ghosh, 2024).

Chicken and duck population counts were sourced directly from the Gridded Livestock of the World dataset (GLW3) (Gilbert et al., 2018). Raw bioclimatic variables were each obtained for each day within the most recent year of data availability (2018) from the ERA5-Land hourly dataset from the Copernicus Climate Data Store (Muñoz Sabater, 2019), queried via the European Centre for Medium-Range Weather Forecasts API (ECMWF) using the R package `ecmfwr`, v2.0.3 (Hufkens et al., 2019). We extracted midday relative humidity, midday temperature, and diurnal temperature range (each measured at 2m elevation) before calculating means for each geolocation-calendar date sampling instance over the 30-day period prior to calendar date. We also extracted and calculated total precipitation and minimum elevation of zero-degree isotherm for each sampling instance over a similar 30-day prior period.

Since several sample geolocations were in close proximity, we used hierarchical clustering to define cluster to avoid simultaneously training and testing on adjacent sites, setting the proximity threshold for cluster membership at 0.15 decimal degrees. This reduced 46 unique geolocations to 25 clusters. When finalised models were applied to predict across a spatial grid spanning the complete area of Egypt, all variables were assigned to this grid based on bilinear interpolation to map to a 0.1-degree resolution except for land use category, which was assigned by modal average.

References for Supplementary Methods

Didan, K., 2022. MODIS/Terra Vegetation Indices 16-Day L3 Global 250m SIN Grid V061.

Friedl, M., Sulla-Menashe, D., 2022. MODIS/Terra+Aqua Land Cover Type Yearly L3 Global 500m SIN Grid V061.

Gilbert, M., Nicolas, G., Cinardi, G., Van Boeckel, T.P., Vanwambeke, S.O., Wint, G.R.W., Robinson, T.P., 2018. Global distribution data for cattle, buffaloes, horses, sheep, goats, pigs, chickens and ducks in 2010. Sci Data 5, 180227. https://doi.org/10.1038/sdata.2018.227

Hassan KE, Ahrens AK, Ali A, El-Kady MF, Hafez HM et al. Improved Subtyping of Avian Influenza Viruses Using an RT-qPCR-Based Low Density Array: 'Riems Influenza a Typing Array', Version 2 (RITA-2). Viruses 2022;14(2).

Hayes, S., Hilton, J., Mould-Quevedo, J., Donnelly, C.A., Baylis, M., Brierley, L., 2025. Ecology and environment predict spatially stratified risk of H5 highly pathogenic avian influenza clade 2.3.4.4b in wild birds across Europe. Sci Rep 16, 997. https://doi.org/10.1038/s41598-025-30651-9

Hijmans, R.J., Barbosa, M., Ghosh, A., Mandel, A., 2024. geodata: Download Geographic Data.

Hijmans, R.J., Ghosh, A., 2024. luna: Tools for Satellite Remote Sensing (Earth Observation) Data Processing.

Hufkens, K., Stauffer, R., Campitelli, E., 2019. The ecwmfr package: an interface to ECMWF API endpoints.

Jarvis, A., Reuter, H.I., Nelson, A., Guevara, E., 2008. Hole-filed seamless SRTM data [WWW Document]. International Centre for Tropical Agriculture (CIAT). URL https://srtm.csi.cgiar.org. (accessed 11.16.25).

Lehner, B., Döll, P., 2004. Development and validation of a global database of lakes, reservoirs and wetlands. Journal of Hydrology 296, 1–22. https://doi.org/10.1016/j.jhydrol.2004.03.028

Massicotte, P., South, A., 2025. rnaturalearth: World Map Data from Natural Earth.

Muñoz Sabater, J., 2019. ERA5-Land hourly data from 1950 to present. https://doi.org/10.24381/cds.e2161bac

Spackman E, Senne DA, Bulaga LL, Myers TJ, Perdue ML et al. Development of real-time RT-PCR for the detection of avian influenza virus. Avian diseases 2003;47(3 Suppl):1079-1082.
